# Implications of bio-efficacy and persistence of insecticides when indoor residual spraying and long-lasting insecticide nets are combined for malaria prevention

**DOI:** 10.1186/1475-2875-11-378

**Published:** 2012-11-19

**Authors:** Fredros O Okumu, Beatrice Chipwaza, Edith P Madumla, Edgar Mbeyela, Geoffrey Lingamba, Jason Moore, Alex J Ntamatungro, Deo R Kavishe, Sarah J Moore

**Affiliations:** 1 Ifakara Health Institute, Environmental Health and Ecological Sciences Thematic Group, P.O Box 53, Ifakara, Tanzania; 2 Department of Disease Control, London School of Hygiene and Tropical Medicine, Keppel Street, London, WC1E 7HT, UK; 3 Nelson Mandela African Institute of Science and Technology, School of Life Sciences, P. O Box 447, Arusha, Tanzania

## Abstract

**Background:**

Bio-efficacy and residual activity of insecticides used for indoor residual spraying (IRS) and long-lasting insecticide nets (LLINs) were assessed against laboratory-reared and wild populations of the malaria vector, *Anopheles arabiensis* in south eastern Tanzania. Implications of the findings are examined in the context of potential synergies and redundancies where IRS and LLINs are combined.

**Methods:**

Bioassays were conducted monthly for six months on three LLIN types (Olyset® PermaNet 2.0®,and Icon Life®) and three IRS treatments (2 g/m^2^ pirimiphos-methyl, 2 g/m^2^ DDT and 0.03 g/m^2^ lambda-cyhalothrin, sprayed on mud walls and palm ceilings of experimental huts). Tests used susceptible laboratory-reared *An. arabiensis* exposed in cones (nets and IRS) or wire balls (nets only). Susceptibility of wild populations was assessed using WHO diagnostic concentrations and PCR for knock-down resistance (*kdr*) genes.

**Results:**

IRS treatments killed ≥ 85% of mosquitoes exposed on palm ceilings and ≥ 90% of those exposed on mud walls, but up to 50% of this toxicity decayed within 1–3 months, except for DDT. By 6th month, only 7.5%, 42.5% and 30.0% of mosquitoes died when exposed to ceilings sprayed with pirimiphos-methyl, DDT or lambda-cyhalothrin respectively, while 12.5%, 36.0% and 27.5% died after exposure to mud walls sprayed with the same insecticides. In wire-ball assays, mortality decreased from 98.1% in 1st month to 92.6% in 6th month in tests on PermaNet 2.0®, from 100% to 61.1% on Icon Life® and from 93.2% to 33.3% on Olyset® nets. In cone bioassays, mortality reduced from 92.8% in 1st month to 83.3% in 6th month on PermaNet 2.0®, from 96.9% to 43.80% on Icon Life® and from 85.6% to 14.6% on Olyset®. Wild *An*. *arabiensis* were 100% susceptible to DDT, 95.8% to deltamethrin, 90.2% to lambda cyhalothrin and 95.2% susceptible to permethrin. No *kdr* gene mutations were detected.

**Conclusions:**

In bioassays where sufficient contact with treated surfaces is assured, LLINs and IRS kill high proportions of susceptible *An*. *arabiensis* mosquitoes, though these efficacies decay gradually for LLINs and rapidly for IRS. It is, therefore, important to always add intact nets in sprayed houses, guaranteeing protection even after the IRS decays, and to ensure accurate timing, quality control and regular re-spraying in IRS programmes. By contrast, adding IRS in houses with intact LLINs is unlikely to improve protection relative to LLINs alone, since there is no guarantee that unfed vectors would rest long enough on the sprayed surfaces, and because of the rapid IRS decay. However, there is need to clarify these effects using data from observations of free flying mosquitoes in huts. Physiological susceptibility of *An*. *arabiensis* in the area remains 100% against DDT, but is slightly reduced against pyrethroids, necessitating caution over possible spread of resistance. The loss of LLIN toxicity, particularly Olyset® nets suggests that protection offered by these nets against *An*. *arabiensis* may be primarily due to physical bite prevention rather than insecticidal efficacy.

## Background

Decisions to use indoor residual spraying (IRS), long-lasting insecticide nets (LLINs) or the two methods together for malaria vector control are usually made based on proven protective efficacy of the interventions, an understanding of existing epidemiological conditions, and the operational or logistical requirements associated with the interventions [1]. Protective efficacy is itself a function of the behaviour of local mosquito populations [2] and susceptibility of these vectors to those insecticides used for the ITNs or IRS [3].

An increasingly important question to vector control specialists today is whether there are any synergies when LLINs and IRS are combined in the same households [4-6]. Besides, it is also not clear how the different modes of action and other characteristics, such as bio-efficacy and residual activity, of candidate insecticides would affect outcomes of such LLIN-IRS combinations. Another growing concern is the question of whether LLINs and IRS are actually suitable for controlling *Anopheles arabiensis* mosquitoes, which is increasingly becoming the predominant vector in many parts of Africa. There is evidence that *An*. *arabiensis* mosquitoes entering human occupied huts where indoor insecticidal interventions are used, suffer lower mortalities than their sibling species, *Anopheles gambiae sensu stricto*[7]. Moreover, where insecticidal nets have been used for several years, populations of *An*. *arabiensis* are often less reduced than *An*. *gambiae* s.s or *Anopheles funestus* s.s [8-10]. Therefore, in sub-Saharan Africa where residual transmission is maintained primarily by *An*. *arabiensis*, IRS and LLINs, or even combinations of the two methods could have a limited impact, other than the physical barrier provided by intact bed nets against mosquito bites.

An earlier study in south eastern Tanzania evaluated three different insecticides approved by WHO for use in IRS campaigns, i.e. lambda cyhalothrin (a synthetic pyrethroid), pirimiphos methyl (an organophosphate) and DDT (an organochloride), and also three types of LLINs, i.e. Olyset® (a permethrin-impregnated net), PermaNet 2.0® (a deltamethrin-coated net) and Icon Life® (a deltamethrin-impregnated net, similar in properties to the one marketed as NetProtect®). The aim of that study, which was conducted using experimental huts, had been to determine if there can be any additional benefit of combining LLINs with IRS as opposed to using either of the methods alone (Okumu et al., unpublished). This report contains details of a complementary study conducted to assess the bio-efficacy and residual activity of the same LLINs and IRS, against *An*. *arabiensis* mosquitoes.

The aim of this study was to examine implications of insecticidal properties, when deciding whether to combine LLINs and IRS; for example by providing evidence on the need for temporal overlaps to counter problems of reduced efficacy overtime. Moreover, the study would help determine rates at which the insecticidal efficacy of the different LLINs and IRS decay. Finally, it would provide evidence on whether the low mortalities increasingly being observed experimental hut studies (including our parallel LLIN-IRS combination study) are due to reduced susceptibility to insecticides, rather than other factors such as behaviour of the vectors, which could lead to lower contact rates with treated surfaces.

## Methods

### Study area

The study was conducted in Lupiro village (8.385^o^S and 36.670°E) in Ulanga District, south-eastern Tanzania. The village lies 270 m above sea level on the Kilombero river valley, and is 26 km south of Ifakara town, where Ifakara Health Institute (IHI) is located. It borders many small contiguous and perennially swampy rice fields to the northern and eastern sides. The annual rainfall is 1,200-1,800 mm, while temperatures range between 20°C and 33°C. Composition of malaria vector populations (which previously included a mixture of *An*. *gambiae s*.*s*, *An*. *arabiensis* and *An*. *funestus*) has shifted dramatically in recent years, most likely because of high ITN coverage [8], so that today, the most abundant vector is *An*. *arabiensis*, constituting > 97% of the *An*. *gambiae* complex species [11,12]. *Anopheles arabiensis* and *An*. *funestus* species are now the main contributors to malaria transmission in the area.

### Mosquitoes

The mosquitoes used for this study were either: 1) wild female *An*. *arabiensis* mosquitoes caught inside experimental huts constructed in the study village, or 2) wild mosquitoes caught using light traps set next to human occupied bed nets inside local houses, or 3) mosquitoes from a new mosquito colony, established using offspring from blood fed *An*. *arabiensis* mosquitoes collected from local human houses using indoor resting collections in the same area.

The colony was established as follows: resting blood fed *Anopheles gambiae* complex mosquitoes were collected from local houses and were kept in separate water filled vials and left to lay eggs, after which adults were identified by polymerase chain reaction (PCR), to distinguish between *An*. *arabiensis* and *An*. *gambiae s*.*s*[13], the two sibling species of *An*. *gambiae* complex found in the study area. Using the eggs obtained from *An*. *arabiensis*, an insectary colony was established and maintained in a semi-field system inside a screen house at the Ifakara Health Institute [14], to provide mosquitoes for bioassays. The larvae here where regularly fed on ground fish food and adult mosquitoes maintained on 10% sugar solution, at temperatures of 28 - 29°C and 70-80% relative humidity. For purposes of the experiments, we used unfed 2–8 days old nulliparous female mosquitoes.

### LLINs and IRS compounds

Four net types (3 LLINs and 1 non-insecticidal net as the control) and three IRS insecticides of different classes were used. The LLINs included Olyset® nets (manufactured by A-Z, Tanzania, but purchased from local vendors), PermaNet 2.0® nets (Vastergaard, Switzerland) and Icon Life® nets, which has similar specifications as the one marketed under the brand name, NetProtect® (Bestnet Europe ltd, Denmark). Olyset® nets are made of polyethylene netting (150 denier), impregnated during manufacture with synthetic permethrin at 2% w/w (equivalent to 1000 mg of active ingredient/m^2^). PermaNet 2.0® is a 100%-polyester net (100 denier), coated with 55-62 mg of synthetic deltamethrin/m^2^, resulting in insecticide concentrations of approximately 0.14% w/w. Icon Life® is a polyethylene net, impregnated during manufacture with synthetic deltamethrin at 0.2% w/w (approximately 65 mg of active ingredient/m^2^).

The IRS treatments included an organochlorine, i.e. 2 g/m^2^ DDT wettable powder (AVIMA, South Africa), a synthetic pyrethroid, i.e. 0.03 g/m^2^ lambda-cyhalothrin capsule suspension (Syngenta, Switzerland), and an organophosphate, i.e. 2 g/m^2^ pirimiphos-methyl emulsified concentrate, also known as actellic (Syngenta, Switzerland). The insecticides had been sprayed on walls and ceilings of experimental huts using Hudson sprayers according to standard WHOPES guidelines [15], and leaving some huts unsprayed to be used as controls. The walls of these experimental huts had been plastered using local mud, which locals use for house building because of its high clay content, while the ceilings were made of palm woven mats locally known as *Mikeka*.

These candidate IRS compounds and all the LLINs have either full or interim approval from WHO, and represent a diversity of common insecticides applicable for mosquito control [16].

### Assessment of residual activity of the IRS insecticides and LLINs

Based on WHO guidelines for testing mosquito adulticides [17], bioassays were conducted *in situ* to examine residual activity of the insecticides in the bed nets, and on the hut walls and ceilings within the experimental huts, at monthly intervals for up to six months. This duration was determined by the fact this study was embedded onto an ongoing experimental hut evaluation of combinations of LLINs, with IRS products that have residual activity of 2–3 months (pirimiphos methyl), 3–6 months (lambda cyhalothrin), or >6 months (DDT), according to WHO recommendations [18].

#### Residual efficacy of bed nets

Cone bioassays and wire ball tests [17] were conducted on newly unbundled nets, and thereafter repeated once every month, for the six months period during which the LLIN/IRS experimental hut study was conducted. Cones and wire ball assays are both applicable for residual efficacy testing but have the following differences: in the cone assays, mosquitoes are exposed by enclosing them in close proximity to test surfaces using plastic cones. This method was used on spread-out net surfaces, walls and ceilings. By contrast, the wire ball method involves using two intersecting circular frames of wire, each measuring 15 cm diameter, around which the test nets are wrapped to form a netting ball. This method can be used on nets but not on walls or ceilings [17]. The cone and wire frame bioassays were conducted on nets, which were being used by volunteers sleeping in experimental huts, without having to cut off net pieces.

Batches of five mosquitoes (for cone tests) or 11 mosquitoes (for wire ball tests) were exposed for three minutes on each of the five sides of the nets as described in the WHO guidelines [17]. Ten to thirty batches were used for the cone tests and five to fifteen batches for the wire ball tests per month per net type (Additional file 1). The mosquitoes were all 2–8 days old nulliparous females obtained from the specially established colony described above. After exposure, the mosquitoes were transferred to small netting cages (15cm×15cm×15cm) and the number of mosquitoes knocked down within 60 minutes was recorded. The mosquitoes were provided with 10% glucose solution and kept inside a holding room at the study site. Mean indoor temperatures inside the holding room were 29.1°C ± 3.0°C during the day and 26.7°C ± 2.3°C at night, while mean relative humidity was 70.6% ± 17.9% during the day and 75.7% ± 13.7% at night.

The mosquitoes were monitored for 24 hours, after which the numbers of those that survived or died were recorded. Controls, consisting of non-insecticidal mosquito nets, were included alongside each of the assays, and up to four different nets of each type were tested for each replicate.

#### Residual efficacy of IRS

Batches of 10 nulliparous females were introduced into the WHO cones and exposed for 30 minutes on randomly selected sites of each of the four walls of each hut and also on two randomly selected positions on the ceilings of the huts. Eight batches were tested per IRS treatment (Additional file 1). The mosquitoes were monitored for 24 hours as above and mortality recorded. The first of these bioassays on walls and ceilings were done in freshly sprayed experimental huts, two days after the spraying. The bioassays were then repeated once every month for the six-month duration of the LLIN/IRS hut study. Controls, which consisted of unsprayed hut walls and ceilings, were included in each of these assays.

A similar set of bioassays was performed on separate wooden panels (1 m^2^ each), lined with either mud or *Mikeka* to mimic the mud walls and palm ceilings of respectively. The panels had been treated with insecticides the same way as the experimental huts, by attaching them onto the inside surfaces of the door shutters, so that they were sprayed at the same time when the huts were being sprayed. These panels were kept inside the same experimental huts so as to ensure they remained under similar environmental conditions as the sprayed walls and ceilings, for as long as the hut study lasted. However, unlike in the experimental hut bioassays, which were conducted either on vertical surfaces (in the case of sprayed walls) or downward facing horizontal surfaces (in case of ceilings), all assays on the wooden panels were conducted with the panels kept on a flat horizontal surface. There were two mud panels and two *Mikeka* panels sprayed with each of the test IRS compounds. Ten mosquitoes were exposed on four different spots per panel, so that a total of 80 mosquitoes were tested per treatment per surface per month. Controls used here consisted of unsprayed *Mikeka* and or unsprayed mud panels.

### Assessment of susceptibility of local malaria vectors to insecticides used for IRS and LLINs

Given that an earlier study had shown low percentage mortalities even among mosquitoes caught in experimental huts where insecticidal interventions were being used, it was important to assess the possibility of resistance against the insecticides being used. Adult mosquitoes were collected using interception exit traps attached to experimental huts, inside which adult male volunteers slept under intact non-insecticidal nets. These experimental huts and the traps have previously been described elsewhere [19]. For this specific purpose, we used huts that were not previously sprayed with any insecticide. Mosquitoes collected from the huts were provided with 10% sugar solution and maintained under ambient shade conditions in the holding room at our study site, for up to five hours before being used. The mosquitoes were identified morphologically to select *An*. *gambiae* s.l females, which were then subjected to standard WHO insecticide-susceptibility bioassays [20]. The most recent molecular analyses of *An*. *gambiae* mosquitoes from this study village have consistently shown that more than 97% are *An*. *arabiensis*[11,12], therefore, the mosquitoes are hereafter refer to simply as *An*. *arabiensis*.

The insecticide-susceptibility bioassays [20] were performed by exposing the female *An*. *arabiensis* mosquitoes to filter papers impregnated with diagnostic concentrations of the candidate insecticides as follows: deltamethrin (0.05%), permethrin (0.75%), lambda cyhalothrin (0.05%), dieldrin (0.4%) and DDT (4%). The assays were performed at near-room temperature conditions (25 ± 2°C), making sure that the exposure tubes are always held vertically. All the insecticide-impregnated papers, the papers used as controls and all the insecticide-testing tubes were supplied by the Vector Control Research Unit, Universiti Sains Malaysia and were stored under refrigeration at 4°C until use.

In each test 21 to 25 mosquitoes were exposed to the insecticide-impregnated papers for up to 60 minutes in tubes lined with the respective insecticide impregnated papers. During exposure the number of mosquitoes knocked down in each tube was recorded after 10, 15, 20, 30, 40, 50 and 60 minutes. After the 60 minutes exposure, mosquitoes were transferred into clean holding tubes and kept for 24 hours in the holding room, during which time they were provided with 10% sugar solution. Where no knock-down was observed within the initial 60 minutes of exposure, the mosquitoes were transferred from the insecticidal test tubes to clean holding tubes and observed for an additional 20 minutes.

Mortality was monitored and recorded after the 24 hour holding period. Tests included a maximum of 125 mosquitoes per insecticide i.e. five replicates with a maximum of 25 mosquitoes each. Since the daily collections could not yield enough *An*. *arabiensis* females to conduct all the assays at the same time, the replicate tests were performed in consecutive days, making sure we had one control each day.

### Testing for presence of knock down resistance (kdr)-gene mutation in the local mosquito population

The majority of the interventions that we tested in our experimental hut study were pyrethroid based (i.e. Olyset® nets, PermaNet 2.0® nets, Icon Life® nets, and IRS with lambda cyhalothrin). However, since we also tested DDT for IRS, one of the major concerns was possibility that if the low mortalities that were being observed in the huts were due to physiological insecticide resistance, the most likely cause would be presence of knock-down resistance (*kdr*) alleles, which is usually associated with cross-resistance between pyrethroids and DDT [21]. Therefore, molecular analysis was performed with the aim of detecting *kdr* presence among the *An*. *arabiensis* mosquitoes originating from the study area.

Four different groups of mosquitoes were included for the *kdr* analysis, namely: 1) wild *An*. *arabiensis* mosquitoes collected using CDC-light traps from local houses in the same study village where the LLIN/IRS experimental hut study was being conducted: 2) wild *An*. *arabiensis* mosquitoes collected inside the experimental huts used in the LLIN/IRS study, 3) mosquitoes originating from the *An*. *arabiensis* colony that we established using mosquitoes originally collected from the same study village, as described above and 4) mosquitoes which had survived the WHO bioassays performed on the insecticide-sprayed walls, sprayed ceilings and the nets, also as described above. Courtesy of Dr. Raphael N’Guessan of Centre de Recherche Entomologique, Cotonou, Benin (CREC), positive controls were obtained from an area in Benin, where *kdr* allele frequency has been consistently shown to be greater than 95% in recent years [22]. The detection of *kdr* using PCR was performed at Ifakara Health Institute, Tanzania. The protocol adapted was originally developed by Martinez-Torres et al. [23] for detection of both L1014S *kdr* allele (mutation commonly found in East Africa [24,25]) and L1014F *kdr* allele (mutation common in West Africa [23,26]).

### Data analysis

The mortality of mosquitoes in the different bioassays was calculated as a proportion of the total number exposed to each chemical. Abbot’s formula [27] was used to correct the mortality in all tests where the control mortality was higher than 5% as follows: Corrected mortality = [(% mortality in treatment – % mortality in control)/(100 - % mortality in control)] × 100. Where mortality was greater than 10% in the controls, the assay data was discarded. In the susceptibility tests, percentage knock-down was also calculated for each of the time periods when the mosquitoes were observed.

### Molecular distinction of *An*. *gambiae* complex sibling species

A sub-sample of the female *An*. *gambiae* s.l mosquitoes collected in the experimental huts and in the local houses for the bioassays, was examined by polymerase chain reaction (PCR), to determine proportions of *An*. *gambiae s*.*s*. and *An arabiensis* sibling species [13]. All the wild mosquitoes subjected to *kdr* examination were also subjected to the PCR for species identification.

### Protection of participants and ethical approval

Human participants in this study included the volunteers who slept in experimental huts as baits, during the time when adult mosquitoes were being collected for use in the insecticide susceptibility tests. Participation of volunteers in these experiments was voluntary, even though the volunteers received compensation for their time. After full explanation of purpose and requirements of the studies, written informed consent was sought from each volunteer prior to the start of all experiments. While inside the experimental huts, the volunteers slept under intact bed nets as a basic protection against mosquito bites and were also provided with long sleeved, hooded jackets to provide additional protection from bites, whenever the volunteers stepped outside the nets to collect mosquitoes from interception exit traps attached to the huts [19]. In addition, the volunteers were provided with access to diagnosis for malaria parasites using rapid diagnostic test kits, and treatment with the current first-line malaria drug (artemether-lumefantrine) in case they had malaria. Ethical approval for this work was granted by the Institutional Review Board of the Ifakara Health Institute (IHRDC/IRB/No.A019), the Tanzania National Institute of Medical Research (NIMR/HQ/R.8aNo1.W710) and the London School of Hygiene and Tropical Medicine (Ethics Clearance No. 5552).

## Results

### Residual activity of candidate insecticidal application: results of monthly bioassays

**Figure 1:**
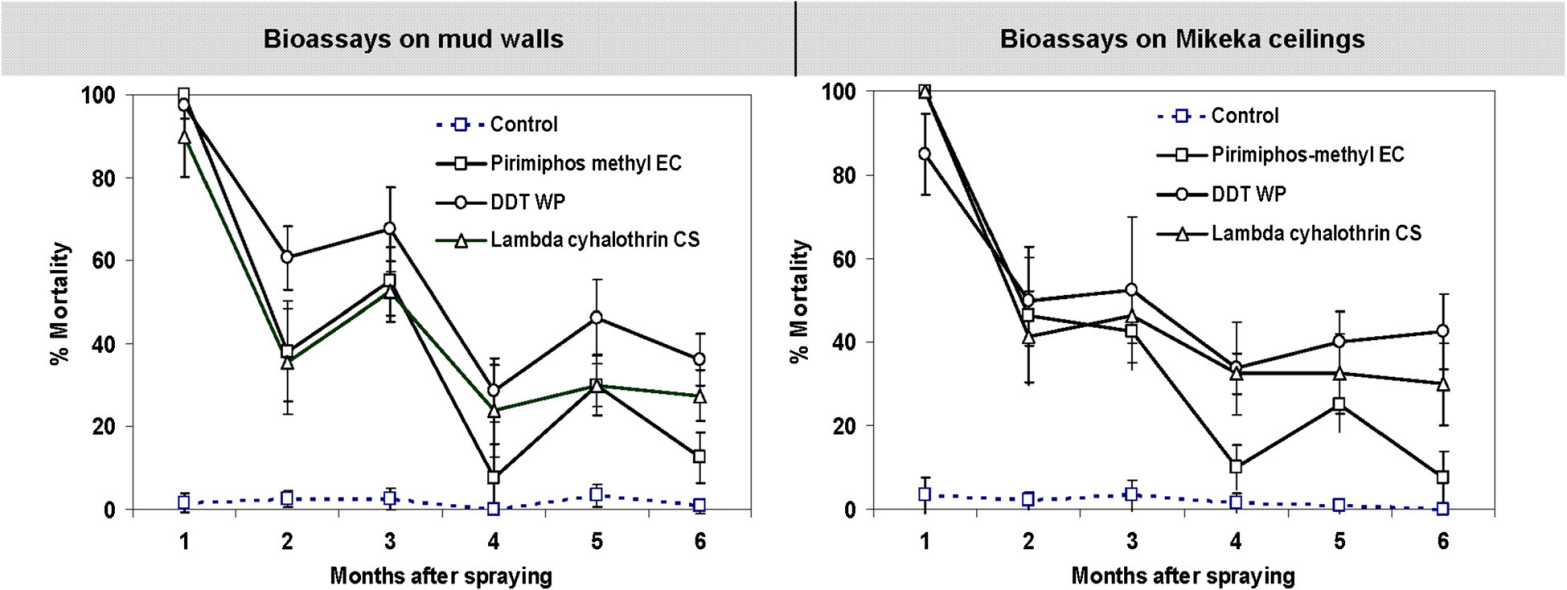
**Results of monthly bioassays showing residual activity of various IRS compounds sprayed on mud walls and *Mikeka *ceilings of experimental huts.** The controls in this assays consisted of bioassays conducted walls and ceilings of unsprayed experimental huts.

**Figure 2:**
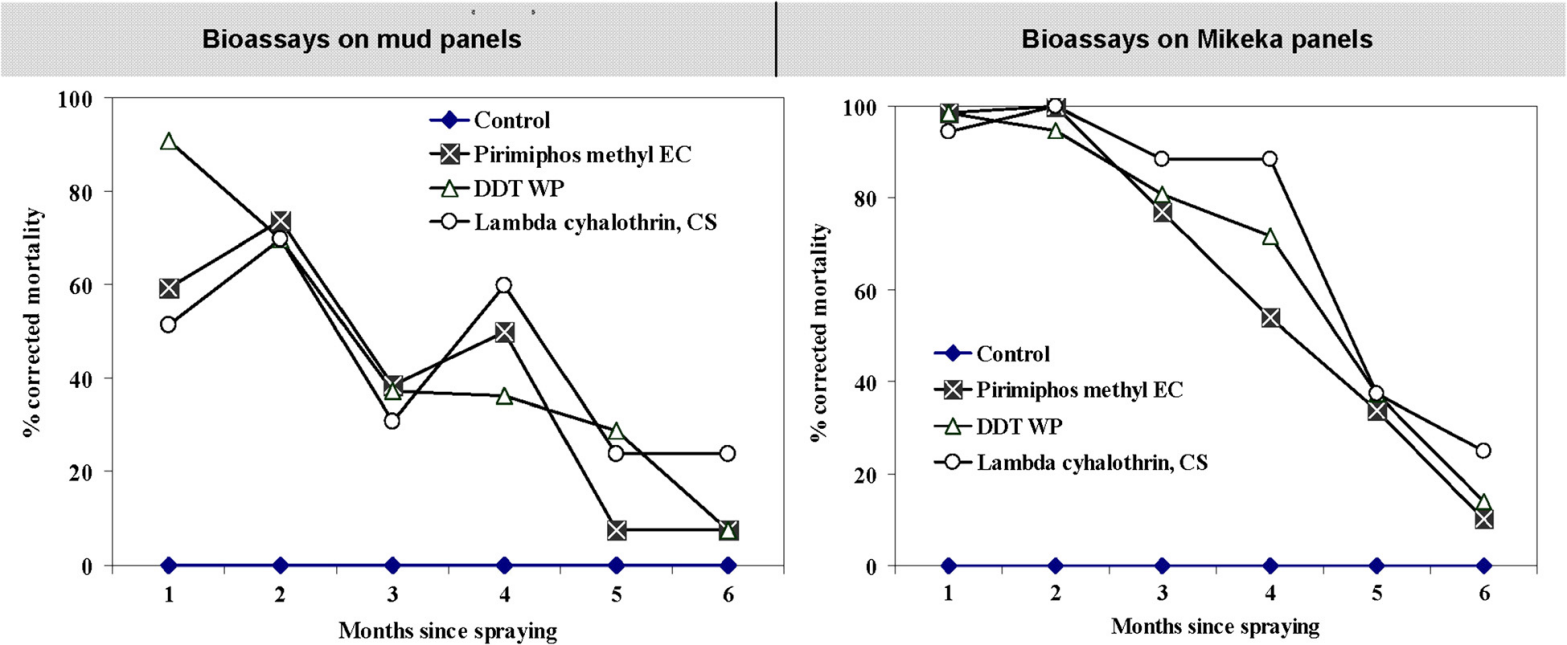
**Results of monthly bioassays showing residual activity of various IRS compounds sprayed on panels (1 sq metre each) lined with *Mikeka *(i.e. *Mikeka *panels) or mud (i.e. mud panels).** The data were corrected using Abbot’s formula [27] to account for unexpectedly high mortalities in the controls. The controls consisted of panels lined with unsprayed mud or *Mikeka.*

**Figure 3:**
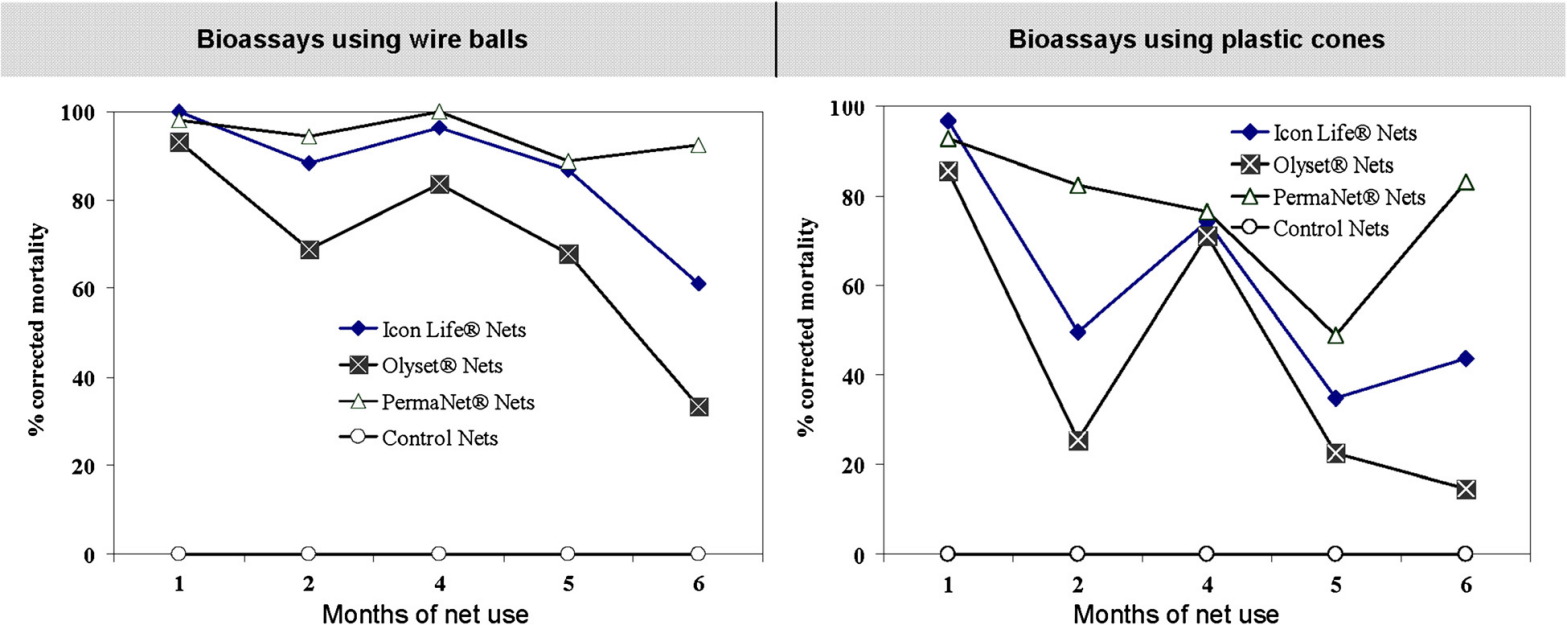
**Results of monthly bioassays showing residual activity of various LLINs when tested using either the standard WHO cone assays or the wire ball method (two intersecting wire cycles, each measuring 15 cm diameter, around which the test net is wrapped to form a netting ball).** The controls in these assays consisted of a non-insecticidal mosquito net. No assays were conducted on the third month due to lack of mosquitoes.

Figures 1, 2, 3 show residual activities of insecticides sprayed on mud walls and ceilings of experimental huts, and the mud and *Mikeka* panels, as well as activity of the LLINs on *An*. *arabiensis* mosquitoes over a period of six months. Additional data including total numbers of mosquitoes exposed per test is provided in Additional file 1. During the first month of spraying, 100% of mosquitoes exposed to *Mikeka* ceilings sprayed with either pirimiphos methyl or lambda cyhalothrin died, whereas only 85% of those exposed to DDT-sprayed ceilings died. On mud walls sprayed with the same chemicals, we observed 100%, 90.0% and 97.5% mortalities respectively during the first month.

Activity of the IRS declined significantly within just two months, so that by the third month, pirimiphos methyl killed only 42.5% of mosquitoes exposed to the treated ceilings and only 55.0% of those exposed to treated walls. Lambda cyhalothrin on the other hand killed only 46.3% on ceilings and 52.5% on walls. By month 6, pirimiphos methyl had nearly entirely decayed, killing only 7.5% of *An*. *arabiensis* exposed to the sprayed ceilings and only 27.5% of those exposed to sprayed walls. By this time, lambda cyhalothrin was now killing only 30.0% on ceilings and 27.5% on walls. The decay of DDT on either of the surfaces was however relatively much slower compared to the other two IRS compounds, and by the sixth month, it was still killing 42.5% of mosquitoes exposed to sprayed ceilings, and 36.3% of those exposed to sprayed walls (Figure 1).

The additional set of data obtained from bioassays on sprayed mud and mikeka panels depict a similar insecticide decay pattern (Figure 1), except that the insecticides sprayed on the *mikeka* panels remained effective for slightly longer than the sprays on the *mikeka* ceilings. Nevertheless, these panel assays also showed that by the sixth month, most of the insecticidal activity had vanished from both mud and *Mikeka* surfaces sprayed with all the candidate insecticides (Figure 2).

Results of bioassays conducted on the different LLINs are shown in Figure 3. This data was adjusted using Abbot’s formula [27], to account for the unexpectedly high mortalities in the controls, which was higher than 5% in some months. While all the net types generally performed better (i.e. killed more mosquitoes) on wire frame assays than on the cone assays, it was surprising that their activity rapidly deteriorated by the second month of use, relative to new unused nets. For example, Olyset® nets killed only 68.9% of *An*. *arabiensis* mosquitoes exposed in wire ball assays and only 25.5% of those exposed in cone assays during the second month bioassays (Figure 3). Only PermaNet2.0® nets retained mosquitocidal efficacy above 80% by the sixth month of net use (killing 92.6% on wire ball tests and 83.3% on cone assays). All the LLINs however retained very high knock-down rates (> 90% in wire ball tests and >80% in cone tests) on the exposed mosquitoes, except Olyset® nets whose knock-down activity reduced to 72.7% on wire ball tests and 62% on cone tests by the sixth month (Additional file 1: Table S3).

### Susceptibility of local *An*. *arabiensis* females to commonly used insecticides

Table 1 below shows the susceptibility status of *An*. *arabiensis* mosquitoes in the study area to the candidate insecticides. Of all the insecticides tested, 100% susceptibility was observed only for DDT. In tests on permethrin, lambda cyhalothrin and deltamethrin, there were signs of insecticide tolerance, with susceptibilities within WHO set range of 80% - 97%, at which resistance should be suspected [20]. However, both DDT (4%) and permethrin (0.75%) elicited very high knock-down rates after 60 minutes of exposure, i.e. 95.2% and 99.2% respectively, while lambda cyhalothrin (0.05%) elicited only 74% knock-down and deltamethrin elicited only 85.9% knock-down after the same period of time. The lowest knock-down rates were observed with 0.4% dieldrin, which after 60 minutes had knocked down only 2.5%. These mosquitoes were monitored for up to 80 minutes as stipulated in the WHO guidelines [20], but the knock-down rate remained merely 26.5% (Table 1).

**Table 1:** **Results of the insecticide susceptibility tests conducted on wild female *Anopheles gambiae s.l *mosquitoes**^**♣**^

**Insecticide tested**	**Total no. exposed**	**Knock Down (KD)**								**Mortality**		
		**10 min**	**15 min**	**20 min**	**30 min**	**40 min**	**50 min**	**60 min**	**%KD 60 min**	**Total no. dead**	**% Dead**	**Class**
Control	123	0	0	0	0	0	1	1	0.8	1.0	0.8	-
4% DDT	124	0	0	7	44	94	114	118	95.2	124.0	100.0	***
0.75% Permethrin	125	0	8	20	71	95	122	124	99.2	119.0	95.2	**
0.05% Lambda cyhalothrin	123	0	0	2	23	60	81	92	74.8	111.0	90.2	**
0.05% Deltamethrin	96	0	5	16	37	60	74	79	85.9	92	95.8	**
0.4% Dieldrin	124	0	0	0	0	0	2	3	2.4	120.0	96.8	**

^♣^ 96% of mosquitoes of this species complex in the study area at the time were identified by PCR as *Anopheles arabiensis.*

Class (Susceptibility grading).

*** The percentage mortality value indicates 100% susceptibility.

** The percentage mortality value indicates insecticide tolerance and possibility of resistance that needs to be confirmed.

### Frequency of knock-down resistance genes among local *An*. *arabiensis* females

Of 522 mosquitoes collected from our experimental huts, there were 383 successful DNA amplifications in PCR assays for both the *kdr* detection and species identification. All of these were determined to be *An*. *arabiensis* and all were *kdr*-negative (100%). A total of 141 *An*. *arabiensis* females obtained from the colony that had been established using wild caught females from the study area were also analysed. Among these mosquitoes, there were 122 successful amplifications in the PCR for detection of *kdr*, all of which were *kdr*-negative (100%). Though, these mosquitoes included those that had survived the standard bioassays on the hut walls and nets, they were all negative for *kdr* alleles. Finally, 43 mosquitoes collected directly from local houses in the study area, using CDC light traps set near occupied bed nets were analysed. In this case there were only 15 successful DNA amplifications in the PCR for both *kdr* detection and species identification, all of which were identified as *An*. *arabiensis* and as being *kdr*-negative.

## Discussion

This study provides essential clues on the bio-efficacy of public health insecticides currently being used for malaria vector control, either singly or in combination, particularly how these insecticides are likely to perform in an area where the primary malaria vectors are *An*. *arabiensis*, that are still susceptible to commonly used insecticides, albeit with clear signs of that this susceptibility is declining. This is an increasingly common scenario in East Africa where high pyrethroid treated bed net coverage has favoured *An*. *arabiensis* over *An*. *gambiae* s.s or *An*. *funestus*[8-10].

Several efforts are now being made to determine whether LLINs and IRS when used together can confer greater benefits than when the two are used alone [1,5,28,29]. Nearly all the studies concluded so far have assumed that LLINs and IRS treatments are not affected by time dependent loss of efficacy, and as such this subject has not been previously examined [1,5,28,29]. For example, in a recent commentary N’Guessan and Rowland [30] noted that a study previously published by Corbel et al. [5] showing no added value of combining LLINs with IRS or insecticidal wall linings, may have been limited by the failure to consider periods when the IRS treatment was actually active. However, Bradley et al. working in Bioko Island, Equatorial Guinea have reported that limited residual life of insecticides can lead to increased malaria risk, but that LLINs can reduce this effect when used alongside IRS [31].

Based on percentage mortalities observed in the bioassays, where contact between mosquitoes and sprayed surfaces is ensured, this study shows that activity of the tested IRS compounds can decline significantly within the first few months after spraying, in many cases becoming ineffective earlier than the time when they would normally be due for re-spraying [18]. According to recommendations made by WHO, DDT should be re-sprayed after every six to 12 months, lambda cyhalothrin every three to six months and pirimiphos methyl, every two to three months [18]. As an example, this study shows that pirimiphos methyl EC caused merely 42.5% mortality on ceilings and only 55.0% on walls by the 3rd month after spraying, down from 100% mortality in the first month. Interestingly, in a concurrent study evaluating LLIN-IRS combinations (Okumu et al., unpublished), pirimiphos methyl was also shown to be the most lethal of all the tested chemicals against the mosquitoes, yet the mean *An*. *arabiensis* mortality over six months was less than 30%. These results may suggest that while standard WHO bioassays have been widely used to assess efficacies of insecticidal interventions over a given period of time, overreliance on those findings without corroborative experimental hut trials, may miss the subtleties associated with insecticide decay or mosquito behaviour in human dwellings, both of which can lead to significantly low mortality rates in human occupied houses.

If a practical situation where malaria control programs can conduct a maximum of two spray rounds per year is considered, it becomes apparent that all the tested IRS compounds in their existing formulations would be minimally appropriate for use in this study area or in areas with similar vector populations and where people use similar construction materials for walls and ceilings, though high toxicity of pirimiphos methyl as observed in our experimental hut study (Okumu et al., unpublished), may make it suitable for short term use, especially against epidemics. The low mortalities observed in the bioassays, would also suggest much poorer performance of the IRS treatments in actual human houses that are correctly using LLINs that prevent mosquitoes feeding, given that unfed mosquitoes may not rest long enough on treated surfaces to pick up lethal doses. Indeed studies have now shown that in areas dominated by *An*. *arabiensis*, as the main malaria vector, the effect of insecticidal interventions is limited [7]. A concurrent study evaluating LLINs and IRS, *An*. *arabiensis* mortalities were consistently low in huts with LLINs or IRS confirming the more limited value of intradomicilliary insecticidal interventions against this species that is more adaptable to feed at times and places where hosts are available than the strictly anthropophagic and endophagic vectors *An*. *gambiae* s.s. and *An*. *funestus* s.s. (Okumu et al., unpublished).

The findings of net bioassays were surprising, especially given that LLINs should retain their insecticidal activity for at least three years and 20 washes [32]. The tests described here depict a very rapid loss of the mosquitocidal activity of the candidate LLINs, especially Olyset® nets; even in the wire ball tests. Whereas LLINs are expected to last at least three years [32], with some such as the Olyset® nets designed to have up to five years of effective life [33], the tests described here show that insecticidal activity can decline significantly within the first few months. For example, Olyset® nets killed only 68.9% of *An*. *arabiensis* mosquitoes exposed in the wire ball assays and only 25.5% of those exposed in the cone assays by the second month of use, and by the sixth month, only 33.3% and 14.6% of the mosquitoes died when exposed to this net in either wire ball tests or cone tests. By the sixth month when this study ended, only the PermaNet 2.0® nets retained toxicity greater than 80%. Despite this rapid decline, it is equally important to note that in this study, we also observed that all candidate LLINs retained high knock-down rates (>90% in wire ball tests and >80% in cone tests) on the exposed mosquitoes, except Olyset® nets whose knock-down activity was slightly reduced to 72.7% on wire ball tests and 62% on cone tests by the sixth month. In some of the bioassays on LLINs and the IRS treated walls and ceilings, there seemed to be unexpectedly low mortalities in month two, such that when the data was plotted, month 2 was slightly out of the general monthly decay trend. Figures 1 and 3 show observed mortality was often higher in month three than month two or in month four than month two. While there is no obvious explanation for this observation, it could possibly be an artifact resulting from mosquito rearing conditions, and is unlikely to change the general inferences from these findings.

One important aspect to consider here is the fact that in this study the nets were not washed at any time during the course of the study, but were instead only dusted occasionally to remove dust. The lack of washing could explain the observation that LLINs such as Olyset® nets, which are known to possess regenerative properties (normally activated after lengthy periods of use, washing or exposure to heat [33,34]), exhibited a decline in activity during this study. Nevertheless, it is reasonable to be concerned about the quality of marketed LLINs, and all stakeholders including, net manufacturers, public health implementers and net users could benefit from improved quality control. Similar losses of LLIN activity have actually been reported also in community level trials, one example being a study in Kenya where 79.6% of Olyset® nets were found to have failed (i.e. having bioassay mortality rates of less than 50% in two consecutive monthly tests) after two years of use, compared to 17.8% of PermaNet nets [35]. The apparent loss of insecticidal activity also may suggest that long before the expected 3–5 years life of LLINs, the only effects of nets that would be left, is the physical barrier effect, where nets work simply by preventing mosquitoes from feeding upon the net occupants rather than killing the mosquitoes. Indeed, in a concurrent evaluation of LLINs and IRS it was determined that intact non-insecticidal nets equally prevent mosquitoes from blood feeding upon net users, just as intact insecticidal nets (Okumu et al., unpublished).

In addition to enabling the assessment of bio-efficacy and residual activity, the wall and ceiling bioassays also highlighted how differences in treatment surface substrates can affect insecticidal efficacy. That is to say, efficacy of active ingredients on mosquitoes is modulated by type of substrate onto which the compound is applied [36]. In this study, two of the IRS insecticides, pirimiphos methyl EC or lambda cyhalothrin CS, killed 100% of mosquitoes exposed to the *Mikeka* ceilings, while DDT WP sprayed on *Mikeka* ceilings killed a modest 85% in the first month. However, on the mud walls sprayed with the same chemicals, we observed 100%, 90.0% and 97.5% mortality respectively in the same period. It seems therefore that, whereas lambda cyhalothrin CS, performed better on ceilings than on mud surfaces, the DDT formulation was clearly better when used on mud walls than when used on *Mikeka* ceilings, from which the water-based wettable powder would more easily have flaked off over time. Similar arguments have been put forth by many previous authors [36-40], and it is thought that such differences are associated with differences in adsorptive properties of the substrates. For instance, mud surfaces can be highly porous and adsorptive to insecticides, and substrates containing alkaline substances may degrade the candidate insecticide faster than substrates without alkaline contents [36,41] In one study where pyrethroids were tested on different substrates, it was found that porous surfaces such as mud can show variability in insecticidal activity, presumably due to absorption of the insecticides, while less porous surfaces such as wood would result in higher insecticidal activity for long periods due to lower rates of insecticide absorption [39]. More recently, Etang et al. [36], also observed variations of insecticide residual bio-efficacy on different types of wall surfaces in Cameroon and, therefore, suggested that local construction materials should be considered when determining lengths of spray cycles as is also demonstrated from this data. Similarly, a recent study in an area in Ghana where malaria vectors were resistant to pyrethroids, organochlorides and carbamates, but not organophosphates showed that pirimiphos methyl formulation, which in this current study was nearly fully deteriorated by the third month, remained effective when sprayed onto cement walls, and can continue to kill *An*. *gambiae* s.l for up to fifteen weeks, matching existing WHO recommendations [42].

With regard to bioassays on nets, it is also clear that the two methods used in this study, i.e. the plastic cone and wire ball method [17], can give different outcomes, and therefore a more careful interpretation is required. The LLINs generally killed more mosquitoes in the wire ball assays than in the cone assays. According to the current LLIN testing guidelines [17], there are two possible alternatives to the WHO cones, which can also be used to assess residual efficacy of insecticidal nets, namely: 1) the use of WHO test tubes (cylinders) lined on the inside with the test nets, and 2) the wire-ball test as used in this study. It is however also suggested that further calibration against the WHO cones is required before the alternative methods can be widely used in testing and evaluation of insecticide for treatment of mosquito nets, an explanation which also suggests an expectation that the two test methods would give different results.

It can be argued that since the wire ball offers no alternative resting sites (unlike in the cone assays, where mosquitoes can occasionally rest on the cotton plug used to seal the insertion hole on top of the cone and, therefore, fail to make adequate contact with the test surfaces), mosquitoes are more likely to be killed in the balls than in the cones. Furthermore, if the active ingredient has irritant properties, which prevent mosquitoes from resting on treated surfaces for extended periods of time, it is possible that exposed mosquitoes would tend to frequently move from point to point making multiple contacts with the treated surfaces and, therefore, leading to greater exposure and higher percentage mortality. In this study however, no mosquitoes were seen avoiding tarsal contact with the netting material during the cone bioassays; neither did we observe many mosquitoes landing on the cotton wool that was used to plug the plastic cones, which would have indicated a significant role of irritancy [43,44]. It is more likely therefore that the reason more mosquitoes died in wire ball assays than the cone assays was the greater total surface area of LLINs and consequently the greater overall quantities of insecticide that these insects were exposed to in the wire balls relative to the cones.

Insecticide susceptibility is usually classified based on proportions of mosquitoes that die when exposed to diagnostic concentrations of test chemicals as follows: 98-100% mortality indicates susceptibility, 80-97% mortality indicates signs of resistance that need to be confirmed and less than 80% mortality indicates that there is insecticide resistance [20]. In a previous nationwide study in Tanzania, where insecticide resistance was assessed in several districts, it was shown that susceptibility of mosquito populations to lambda cyhalothrin, deltamethrin and permethrin had started to diminish in most of the sentinel districts in the country, including Kilombero district, which neighbours Ulanga district where this current study was conducted [45]. In that study, standard WHO insecticide susceptibility tests on *An*. *gambiae* s.l from Kilombero district, showed 93.9% mortality after exposure to 0.05% lambda cyhalothrin, 96% mortality after exposure to 0.75% permethrin and 90.3% mortality after exposure to 0.05% deltamethrin [45]. Results from this current study (Table 1), depict a closely similar outcome two years later, i.e. full susceptibility to DDT, and reduced susceptibility to lambda cyhalothrin (mortality = 90.2%), permethrin (mortality = 95.2%) and deltamethrin (mortality = 95.8%). While the resistance limits in this area have not yet reached a state where vector control interventions such as pyrethroid based LLINs and IRS with DDT would be considered ineffective, the potentially declining susceptibility to common vector control insecticides clearly calls for continued constant monitoring and resistance management planning to be incorporated in any future vector control campaigns.

The good news, however, was that both the bioassays and the molecular analysis conducted to detect *kdr* alleles, confirmed absence of target site resistance to pyrethroids and DDT, which is one of the mechanisms linked to genetic mutations in the *para*-sodium channels in several insects [46]. Pyrethroid-DDT cross-resistance currently presents, what is perhaps the greatest challenge to insecticide based malaria interventions in Africa [47,48]. Susceptibility surveys have thus become standard pre-requisites, providing baseline data on insecticide susceptibility status, to support large scale LLINs and IRS campaigns in Africa [48,49]. Two different *kdr* mutations have been found in the African malaria vector *An*. *gambiae s*.*s*, including one in West Africa, which is caused by a leucine to phenylalanine substitution (L1014F) [23,26] in the genetic sequence coding for the sodium channels, and a different mutation in East Africa, caused by leucine to serine substitution at the same amino acid position (L1014S) [24,25]. Though the *kdr*-detection protocol used in this study could detect either of the two mutations [23], they were not detected in any of the mosquitoes, and instead, there was a 100% *kdr*-negative rate in all the samples. It should be noted however that these tests may not be conclusive as there may be other modes of physiological resistance, which will also need to be investigated in the future.

If the results of this study are interpreted in the context of an increasingly important question of whether there are any added advantages of combining LLINs with IRS, relative to using each individual application separately [4-6,28], they provide evidence to support the need for adding LLINs where IRS is the only existing intervention. Since most of the IRS candidate insecticides decay so quickly, and since it can be difficult to regularly re-spray houses at the frequencies stipulated by WHO [18], addition of LLINs in such houses would provide additional reduction in indoor mosquito biting rates or potentially kill additional mosquitoes, and would add the temporal overlap necessary to protect house occupants during the period after which the IRS is no longer efficacious. Similar suggestions have been made by Bradley et al. who recently attributed increased malaria risk to the time dependent insecticidal decay [31]. On the other hand, to benefit from adding IRS into houses where LLINs are already being used, especially where the predominant vector is *An*. *arabiensis*, it will be necessary to ensure that the IRS campaigns are properly timed, regular, quality controlled and use highly mosquitocidal chemicals, preferably organophosphates or carbamates rather than pyrethroids as in all current LLINs, a strategy which would help mitigate against spread of insecticide resistance [50]. Where IRS treatments significantly deter malaria mosquitoes from entering houses, it may also be important to ensure implementation at consistently high household coverage to ensure that even those mosquitoes that are deterred from one house to not find blood meals in other unprotected households [1].

## Conclusion

It is concluded that the insecticidal efficacy of all the three IRS compounds, DDT WP, lambda cyhalothrin CS and pirimiphos methyl EC, decay rapidly within the first few months after spraying, necessitating that whenever houses are sprayed with the insecticides, LLINs should also be used in those houses to provide the necessary protection from mosquito bites, even after the IRS protection has been lost. Moreover, the IRS operations should be quality-controlled, regularly repeated and properly timed to match any seasonal variations in malaria transmission, and to achieve the desired impact. LLINs also lose their insecticidal efficacy with time, in some cases by up to 50% or more within just six months, though they can continue to directly protect users from mosquito bites as long as they are intact. Indeed, the loss of LLIN toxicity, particularly for Olyset® nets suggests that protection offered by these nets against *An*. *arabiensis* may be primarily from physical bite prevention rather than insecticidal efficacy. It is however unclear whether these LLIN decays would continue where nets are used for longer than six months (as tested in this study), or if the nets are washed regularly.

The *An*. *arabiensis* mosquitoes in this study area are still fully susceptible to DDT and no knock-down resistance genes were detectable in the vector populations. However, the observed tolerance to pyrethroids necessitates caution against possibility of physiological resistance arising and spreading rapidly across the area in the near future. The observed time-dependent decays notwithstanding, the high percentage mortalities of mosquitoes exposed to new treatments or diagnostic concentrations suggests that the extremely low vector mortalities observed in the concurrent experimental huts study (Okumu et al, unpublished) cannot be explained by reduced susceptibility. Instead it may be a result of the vectors encountering bed nets in the huts and failing to feed, thereby exiting the huts without having rested long enough on treated surfaces to pick up lethal doses.

While intact nets are clearly a necessary addition in any insecticide-sprayed houses, these findings suggest that in areas predominated by *An*. *arabiensis*, where transmission is not highly seasonal and LLIN coverage is already high, the addition of IRS may not be an efficient use of resources. Instead, the focus should be on ensuring that LLINs are used widely and correctly, and are replaced regularly. Finally, the data also emphasizes the need for complementary prevention methods that can target the behavioural resilience of vectors such as *An*. *arabiensis*, in communities which already have high LLIN coverage.

## Competing interests

The authors declare that they have no competing interests.

## Authors' contributions

FOO, BC, SJM and JM designed the study. FOO, SJM, EPM, ED, GL, DRK and AJN collected and analysed the data. FOO and SJM drafted the original manuscript. All authors read, corrected and approved the final manuscript before submission.

## Supplementary Material

Additional file 1 **Table S1:** results of monthly bioassays showing residual activity of various IRS compounds sprayed on hut walls and ceilings. **Table S2**: results of monthly bioassays showing residual activity of various IRS compounds sprayed on separate panels made of mud or *Mikeka*. **Table S3**: results of monthly bioassays showing residual activity of various LLINs*. Click here for file
