# Supplementary material for: Implications of bio-efficacy and persistence of insecticides when indoor residual spraying and long-lasting insecticide nets are combined for malaria prevention
Source: Malar J. 2012 Nov 19;11:378. doi: 10.1186/1475-2875-11-378 (PMC3585720; doi:10.1186/1475-2875-11-378)
Supplement: Additional file 1 — Table S1: results of monthly bioassays showing residual activity of various IRS compounds sprayed on hut walls and ceilings. Table S2: results of monthly bioassays showing residual activity of various IRS compounds sprayed on separate panels made of mud or Mikeka. Table S3: results of monthly bioassays showing residual activity of various LLINs*. [file 1475-2875-11-378-S1.doc]

**Okumu et al Supplementary files**

**Table S1:** results of monthly bioassays showing residual activity of various IRS compounds sprayed on hut walls and ceilings.

|  |  | Bioassays on *Mikeka* ceilings | | | Bioassays on mud walls | | |
| --- | --- | --- | --- | --- | --- | --- | --- |
| Insecticide | Months | No. mosquitoes exposed | Number dead | Percentage mortality | No. mosquitoes exposed | Number dead | Percentage mortality |
| Control | 1 | 60 | 2 | 3.3 | 120 | 2 | 1.7 |
|  | 2 | 240 | 5 | 2.1 | 240 | 6 | 2.5 |
|  | 3 | 120 | 4 | 3.3 | 120 | 3 | 2.5 |
|  | 4 | 120 | 2 | 1.7 | 120 | 0 | 0.0 |
|  | 5 | 120 | 1 | 0.8 | 120 | 4 | 3.3 |
|  | 6 | 120 | 0 | 0.0 | 120 | 1 | 0.8 |
| Actellic EC | 1 | 40 | 40 | 100.0 | 80 | 80 | 100.0 |
|  | 2 | 160 | 74 | 46.3 | 160 | 60 | 37.5 |
|  | 3 | 80 | 34 | 42.5 | 80 | 44 | 55.0 |
|  | 4 | 80 | 8 | 10.0 | 80 | 6 | 7.5 |
|  | 5 | 80 | 20 | 25.0 | 80 | 24 | 30.0 |
|  | 6 | 80 | 6 | 7.5 | 80 | 10 | 12.5 |
| DDT WP | 1 | 40 | 34 | 85.0 | 80 | 78 | 97.5 |
|  | 2 | 159 | 79 | 49.7 | 160 | 97 | 60.6 |
|  | 3 | 80 | 42 | 52.5 | 80 | 54 | 67.5 |
|  | 4 | 80 | 27 | 33.8 | 80 | 23 | 28.8 |
|  | 5 | 80 | 32 | 40.0 | 80 | 37 | 46.3 |
|  | 6 | 80 | 34 | 42.5 | 80 | 29 | 36.3 |
| Lambda cyhalothrin, CS | 1 | 40 | 40 | 100.0 | 80 | 72 | 90.0 |
|  | 2 | 160 | 66 | 41.3 | 160 | 57 | 35.6 |
|  | 3 | 80 | 37 | 46.3 | 80 | 42 | 52.5 |
|  | 4 | 80 | 26 | 32.5 | 80 | 19 | 23.8 |
|  | 5 | 80 | 26 | 32.5 | 80 | 24 | 30.0 |
|  | 6 | 80 | 24 | 30.0 | 80 | 22 | 27.5 |

**Table S2:** results of monthly bioassays showing residual activity of various IRS compounds sprayed on separate panels made of mud or *Mikeka*.

|  |  | Bioassays on *Mikeka* panels | | | Bioassays on mud panels | | |
| --- | --- | --- | --- | --- | --- | --- | --- |
| Insecticide used | Months | No. mosquitoes exposed | Number dead | Percentage mortality | No. mosquitoes exposed | Number dead | Percentage mortality |
| Control | 1 | 40 | 4 | 10.0 | 40 | 2 | 5.0 |
|  | 2 | 40 | 2 | 5.0 | 40 | 2 | 5.0 |
|  | 3 | 40 | 1 | 2.5 | 40 | 1 | 2.5 |
|  | 4 | 40 | 1 | 2.5 | 40 | 4 | 10.0 |
|  | 5 | 40 | 0 | 0.0 | 40 | 0 | 0.0 |
|  | 6 | 40 | 0 | 0.0 | 40 | 0 | 0.0 |
| Actellic EC | 1 | 80 | 80 | 100.0 | 80 | 60 | 75.0 |
|  | 2 | 80 | 79 | 98.8 | 80 | 49 | 61.3 |
|  | 3 | 80 | 62 | 77.5 | 80 | 32 | 40.0 |
|  | 4 | 80 | 44 | 55.0 | 80 | 44 | 55.0 |
|  | 5 | 80 | 27 | 33.8 | 80 | 18 | 22.5 |
|  | 6 | 80 | 8 | 10.0 | 80 | 6 | 7.5 |
| DDT WP | 1 | 80 | 79 | 98.8 | 80 | 73 | 91.3 |
|  | 2 | 80 | 76 | 95.0 | 80 | 57 | 71.3 |
|  | 3 | 80 | 65 | 81.3 | 80 | 31 | 38.8 |
|  | 4 | 80 | 58 | 72.5 | 80 | 34 | 42.5 |
|  | 5 | 80 | 30 | 37.5 | 80 | 23 | 28.8 |
|  | 6 | 80 | 11 | 13.8 | 80 | 6 | 7.5 |
| Lambda cyhalothrin, CS | 1 | 80 | 76 | 95.0 | 80 | 43 | 53.8 |
|  | 2 | 80 | 80 | 100.0 | 80 | 57 | 71.3 |
|  | 3 | 80 | 71 | 88.8 | 80 | 26 | 32.5 |
|  | 4 | 80 | 71 | 88.8 | 80 | 51 | 63.8 |
|  | 5 | 80 | 30 | 37.5 | 80 | 23 | 28.8 |
|  | 6 | 80 | 20 | 25.0 | 80 | 19 | 23.8 |

**Table S3:** results of monthly bioassays showing residual activity of various LLINs***.

|  |  | **Bioassays performed using wire balls** | | | | | **Bioassays performed using cones** | | | | |
| --- | --- | --- | --- | --- | --- | --- | --- | --- | --- | --- | --- |
| Net Type | Months | No. Exposed | No. knocked down | No. dead | % KD | % mortality | No. Exposed | No. knocked down | No. dead | % KD | % mortality |
| Control | 1 | 110 | 6 | 7 | 5.5 | 6.4 | 100 | 2 | 3 | 2.0 | 3.0 |
|  | 2 | 165 | 0 | 1 | 0.0 | 0.6 | 150 | 1 | 1 | 0.7 | 0.7 |
|  | 4 | 110 | 3 | 5 | 2.7 | 4.5 | 100 | 3 | 10 | 3.0 | 10.0 |
|  | 5 | 55 | 0 | 2 | 0.0 | 3.6 | 50 | 0 | 1 | 0.0 | 2.0 |
|  | 6 | 55 | 0 | 1 | 0.0 | 1.8 | 50 | 0 | 2 | 0.0 | 4.0 |
| Icon Life® | 1 | 110 | 108 | 110 | 98.2 | 100.0 | 100 | 100 | 97 | 100.0 | 97.0 |
|  | 2 | 165 | 165 | 146 | 100.0 | 88.5 | 150 | 139 | 75 | 92.7 | 50.0 |
|  | 4 | 110 | 110 | 106 | 100.0 | 96.4 | 100 | 95 | 77 | 95.0 | 77.0 |
|  | 5 | 55 | 55 | 48 | 100.0 | 87.3 | 50 | 46 | 18 | 92.0 | 36.0 |
|  | 6 | 55 | 55 | 34 | 100.0 | 61.8 | 50 | 50 | 23 | 100.0 | 46.0 |
| Olyset® | 1 | 110 | 108 | 103 | 98.2 | 93.6 | 100 | 98 | 86 | 98.0 | 86.0 |
|  | 2 | 165 | 165 | 114 | 100.0 | 69.1 | 150 | 104 | 39 | 69.3 | 26.0 |
|  | 4 | 110 | 104 | 93 | 94.5 | 84.5 | 100 | 87 | 74 | 87.0 | 74.0 |
|  | 5 | 55 | 55 | 38 | 100.0 | 69.1 | 50 | 45 | 12 | 90.0 | 24.0 |
|  | 6 | 55 | 40 | 19 | 72.7 | 34.5 | 50 | 31 | 9 | 62.0 | 18.0 |
| PermaNet® | 1 | 110 | 108 | 108 | 98.2 | 98.2 | 100 | 97 | 93 | 97.0 | 93.0 |
|  | 2 | 165 | 165 | 156 | 100.0 | 94.5 | 150 | 150 | 124 | 100.0 | 82.7 |
|  | 4 | 110 | 107 | 110 | 97.3 | 100.0 | 100 | 91 | 79 | 91.0 | 79.0 |
|  | 5 | 55 | 55 | 49 | 100.0 | 89.1 | 50 | 46 | 25 | 92.0 | 50.0 |
|  | 6 | 55 | 55 | 51 | 100.0 | 92.7 | 50 | 50 | 42 | 100.0 | 84.0 |

*The control consists of non insecticidal nets. Knock-down was assessed 60 minutes after exposure while mortality was assessed after 24 hours of exposure. No bioassays were conducted in the third month due to lack of mosquitoes.
